# Extrapulmonary tuberculosis among migrants in Europe, 1995 to 2017

**DOI:** 10.1016/j.cmi.2020.12.006

**Published:** 2021-09

**Authors:** Sally E. Hayward, Kieran Rustage, Laura B. Nellums, Marieke J. van der Werf, Teymur Noori, Delia Boccia, Jon S. Friedland, Sally Hargreaves

**Affiliations:** 1) Institute for Infection and Immunity, St George's, University of London, London, UK; 2) Faculty of Public Health and Policy, London School of Hygiene & Tropical Medicine, London, UK; 3) European Centre for Disease Prevention and Control (ECDC), Solna, Sweden; 4) Division of Epidemiology and Public Health, School of Medicine, University of Nottingham, Nottingham, UK

**Keywords:** Europe, Extrapulmonary tuberculosis, Health services research, Migrant, Tuberculosis

## Abstract

**Objectives:**

The proportion of tuberculosis (TB) cases occurring in migrants in Europe is increasing. Extrapulmonary TB poses challenges in diagnosis and treatment and causes serious morbidity and mortality, yet its extent in migrant populations is unclear. We assessed patterns of extrapulmonary TB in migrants across the European Union (EU)/European Free Trade Association (EFTA). We investigated the proportion of extrapulmonary TB cases among migrants versus non-migrants, and variations by specific site of disease, reporting European region, and migrant region of origin.

**Methods:**

We carried out a cross-sectional secondary database analysis, utilizing 23 years of data collected between 1995 and 2017 from the European Surveillance System of the European Centre for Disease Prevention and Control for 32 EU/EFTA countries.

**Results:**

In total, 1 270 896 TB cases were included, comprising 326 987 migrants (25.7%) and 943 909 non-migrants (74.3%). Of TB cases among migrants, 45.2% (*n* = 147 814) were extrapulmonary compared to 21.7% (*n* = 204 613) among non-migrants (p < 0.001). Lymphatic, bone/joint and peritoneal/digestive TB were more common among migrant than non-migrant extrapulmonary cases. A lower proportion of extrapulmonary TB was seen in Eastern Europe (17.4%, *n* = 98 656 of 566 170) and Southern Europe (29.6%, *n* = 62 481 of 210 828) compared with Western (35.7%, *n* = 89 498 of 250 517) and Northern Europe (41.8%, *n* = 101 792 of 243 381). Migrants from South-East Asia and Sub-Saharan Africa were at highest risk of extrapulmonary disease, with 62.0% (*n* = 55 401 of 89 353) and 54.5% (*n* = 38 327 of 70 378) of cases, respectively, being extrapulmonary.

**Conclusions:**

Among TB cases in the EU/EFTA, extrapulmonary disease is significantly more common in migrants than in non-migrants. There is a need to improve clinical awareness of extrapulmonary TB and to integrate its detection into screening programmes.

## Introduction

The proportion of tuberculosis (TB) cases in migrants has been rising across high-income countries in Europe [1] and the United States of America (USA) [2]. In 2017, migrants comprised 33.1% of TB cases across countries of the European Union/European Economic Area (EU/EEA), and over 70% in parts of Northern and Western Europe [3]. This trend is set to continue, as TB notification rates are falling faster in native-born residents than in those of foreign origin in the EU/EEA [4]. In addition, migrant populations in high-income countries are growing, with 4.4 million people migrating to an EU Member State during 2017 [5].

While TB most commonly affects the lungs, it can present in almost any organ of the body, including the lymph nodes, pleura, central nervous system (CNS), bones and joints, genitourinary tract and gastrointestinal system [6], or disseminated as miliary TB [7]. While not usually transmissible, extrapulmonary TB can cause serious morbidity and mortality. It is more common among immunosuppressed individuals such as those with HIV/AIDS. Extrapulmonary TB poses challenges in diagnosis and treatment due to its wide variety of non-specific clinical presentations [8].

Globally, 15% of the 7 million incident TB cases notified in 2018 were extrapulmonary [9]. Despite this, extrapulmonary TB is rarely specifically incorporated into TB control programmes, including those targeting migrants. Current guidance from the European Centre for Disease Prevention and Control (ECDC) recommends screening for active pulmonary TB using chest x-ray and screening for latent tuberculosis infection (LTBI) using the tuberculin skin test (TST) or an interferon-γ release assay (IGRA) soon after migrants from countries with a high incidence of TB arrive in the EU/EEA [10], with no specific provision for detecting extrapulmonary disease.

Patterns of extrapulmonary TB in migrants are not well understood, despite implications for morbidity and mortality. We aim to assess patterns of extrapulmonary TB in migrants in EU/European Free Trade Association (EFTA) countries through analysis of the ECDC's European Surveillance System (TESSy) database. The specific objectives are to examine whether the proportion of extrapulmonary TB cases is greater in migrants than in non-migrants, which specific sites of extrapulmonary disease are more or less common in migrants, and how the distribution of cases varies by European reporting region and migrants' region of origin.

## Methods

### Data and definitions

We analysed 23 years of data from ECDC's TESSy database for 32 EU/EFTA countries (henceforth ‘Europe’) collected between its inception in 1995 and 2017. Detailed data collection methods and definitions have been described [3]. In brief, designated experts within national surveillance institutes submit case-based data to TESSy, where a TB case is defined following the World Health Organization (WHO) as a bacteriologically confirmed or clinically diagnosed case [11]. Data on all reported cases in Europe between 1995 and 2017 were extracted.

The analysis was restricted to TB cases with known migrant status and TB site. We define a migrant as a person born in, or having citizenship of, a country different from the reporting country. Extrapulmonary TB is classified as a case involving organs or anatomical sites other than the lungs, with or without coexistent lung disease. Specific sites of extrapulmonary TB were grouped as: lymphatic, pleural, bone/joint (including spine), disseminated, genitourinary, peritoneal/digestive, CNS (including meningitis), and other. Region of Europe was defined using the United Nations Geoscheme for Europe: Eastern, Northern, Southern and Western [12]. Region of origin was defined using an adaptation of World Bank regions: Eastern Europe and Central Asia, Europe, North America and Oceania, Latin America and the Caribbean, Middle East and North Africa, South-East Asia, and Sub-Saharan Africa [13]. Definitions are presented in the Supplementary Material Fig. S1.

### Statistical methods

We assessed how key demographic characteristics differed between migrants and non-migrants, using t tests for continuous variables and χ^2^ tests for categorical variables. We compared the difference in the proportion of extrapulmonary TB between migrants and non-migrants using χ^2^ tests. We repeated this analysis in subgroups, firstly dividing the sample into drug-susceptible and multidrug-resistant (MDR) TB (MDR being defined as resistance to at least isoniazid and rifampicin), and secondly dividing the study period in two (1995–2006 and 2007–2017). In addition, we used two-sample tests of proportion to compare the difference in proportion of extrapulmonary TB at a given site between migrants and non-migrants, χ^2^ tests to compare the proportion of TBs that are extrapulmonary between the different regions of Europe, and one-sample tests of proportion to compare the difference in proportion of pulmonary versus extrapulmonary TB in each migrant region of origin. In sensitivity analyses, we repeated these analyses defining extrapulmonary TB as cases involving *only* organs or anatomical sites other than the lungs. A value of p < 0.01 was considered statistically significant. All analyses were conducted using StataSE v15.

### Ethical statement

The study was based on data collected on the basis of statutory notification in each EU/EFTA country and reported anonymously to ECDC per decision No 2119/98/EC of the European Parliament and of the Council.

## Results

### Sample characteristics

**Fig. 1:**
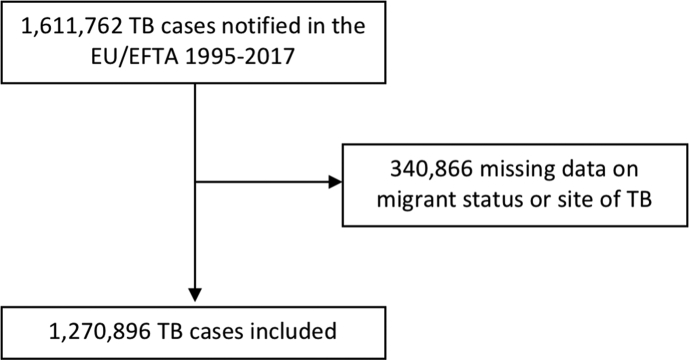
Sample flow chart. EU/EFTA, European Union/European Free Trade Association; TB, tuberculosis.

In total, 1 611 762 TB cases were notified in the EU/EFTA between 1995 and 2017 and reported in TESSy, of which 1 270 896 (78.9%) had data available on migrant status and TB site and were included in the analyses (Fig. 1). The included sample comprises 326 987 migrants (25.7%) and 943 909 non-migrants (74.3%). Migrant regions of origin and destination are shown in the Supplementary Material Fig. S2, and sample characteristics by migrant status are presented in Table 1.

**Table 1:** Characteristics of migrant and non-migrant tuberculosis (TB) cases in the European Union/European Free Trade Association (EU/EFTA), 1995–2017 (*n* = 1 270 896)

	Migrants		Non-migrants		Total	
	n	%	n	%	n	%
Total	326 987	25.7	943 909	74.3	1 270 896	
Age:						
*Mean, SD*	37.9	16.8	47.8	20.3	45.3	19.9
Gender:						
*Female*	130 944	40.1	320 536	34.0	451 480	35.6
*Male*	195 264	59.9	622 902	66.0	818 166	64.4
Region of origin:						
*Eastern Europe and Central Asia*	56 310	21.6	558 595	63.1	614 905	53.6
*Europe, North America and Oceania*	24 392	9.3	326 970	36.9	351 362	30.6
*Latin America and the Caribbean*	11 329	4.3	—		11 330	1.0
*Middle East and North Africa*	9399	3.6	—		9405	0.8
*South-East Asia*	89 303	34.2	—		89 353	7.8
*Sub-Saharan Africa*	70 341	26.9	—		70 378	6.1
Reporting region:						
*Eastern Europe*	4921	1.5	561 249	59.5	566 170	44.6
*Southern Europe*	69 159	21.2	141 669	15.0	210 828	16.6
*Western Europe*	128 360	39.3	122 157	12.9	250 517	19.7
*Northern Europe*	124 547	38.1	118 834	12.6	243 381	19.2
Previous TB diagnosis:						
*Yes*	24 864	10.4	143 771	17.0	168 635	15.5
*No*	213 423	89.6	703 879	83.0	917 302	84.5
Site of TB:						
*Pulmonary*	179 173	54.8	739 296	78.3	918 469	72.3
*Extrapulmonary*^a^	147 814	45.2	204 613	21.7	352 427	27.7

Data reported as: n, % unless otherwise stated. Sample sizes: age, *n* = 1 268 544; gender, *n* = 1 269 646; region of origin, *n* = 1 146 733; reporting region, *n* = 1 270 896; previous TB diagnosis, *n* = 1 085 937; site of TB, *n* = 1 270 896. The difference between migrants and non-migrants for each characteristic is statistically significant at p < 0.001 calculated using t test for continuous variables or χ^2^ for categorical variables: age, t = 251.9; gender, χ^2^ = 4.0 × 10^3^; reporting region, χ^2^ = 3.6 × 10^5^; previous TB diagnosis, χ^2^ = 6.0 × 10^3^; site of TB, χ^2^ = 6.7 × 10^4^.

a Extrapulmonary TB defined as any case of TB involving organs or anatomical sites other than the lungs, with or without co-existent lung disease.

The mean age of included migrants with TB was 38 years compared with 48 years in non-migrants (p < 0.001), and 59.9% of migrants (*n* = 195 264 of 326 208) were male compared with 66.0% of non-migrants (*n* = 622 902 of 943 438) (p < 0.001). Migrant TB cases in the EU/EFTA originate from diverse areas of the world, most commonly South-East Asia (34.2%, *n* = 89 303 of 261 074) and Sub-Saharan Africa (26.9%, *n* = 70 341). The most common countries of origin for migrants with TB are India (*n* = 30 174), Pakistan (*n* = 23 081), Somalia (*n* = 20 453), and Romania (*n* = 14 620). In Eastern and Southern Europe, the majority of TB cases are in non-migrants, whereas in Northern and Western Europe around half are in migrants. Migrants were reported to be less likely than non-migrants to have been previously diagnosed with TB (p < 0.001).

### Extrapulmonary TB in migrants

**Fig. 2:**
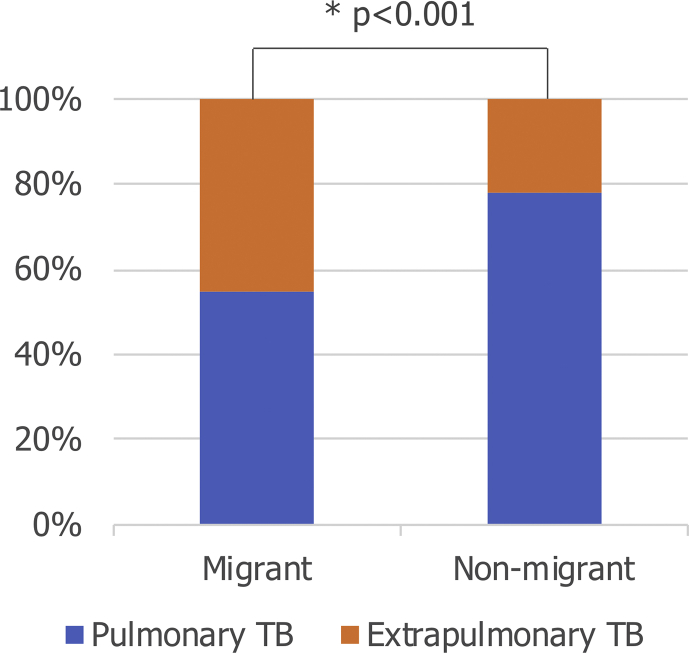
Site of tuberculosis (TB) among migrant and non-migrant TB cases in the European Union/European Free Trade Association (EU/EFTA), 1995–2017 (*n* = 1 270 896). Extrapulmonary TB is defined as any case of TB involving organs or anatomical sites other than the lungs, with or without coexistent lung disease. p value calculated using χ^2^ test (χ^2^ = 6.7 × 10^4^, p < 0.001).

The proportion of TB that is extrapulmonary is significantly greater among migrants than non-migrants (Fig. 2): 45.2% of cases among migrants (*n* = 147 814 of 326 987) were extrapulmonary, compared to 21.7% among non-migrants (*n* = 204 613 of 943 909) (p < 0.001). This pattern is seen in both drug-susceptible and MDR TB (both p < 0.001), and in the earlier (1995–2006) and later (2007–2017) parts of the study period (both p < 0.001) (Supplementary Material Table S1).

Among extrapulmonary TB cases, specific site varies by migrant status (Table 2). For migrants, 47.6% of extrapulmonary TB is lymphatic (*n* = 37 150 of 78 077), compared with 25.3% in non-migrants (*n* = 43 933 of 173 604) (p < 0.001). This varies by country of origin; for example, among migrants from India, 52.4% of extrapulmonary TB is lymphatic (*n* = 2079 of 3971). Conversely, 17.6% of extrapulmonary TB is pleural in migrants (*n* = 13 730 of 78 077) versus 42.1% in non-migrants (*n* = 73 052 of 173 604) (p < 0.001); 24 965 TB cases (4336 migrants and 20 629 non-migrants) were reported to include both pulmonary and pleural involvement. Differences are also seen for bone/joint TB (p < 0.001) and peritoneal/digestive TB (p < 0.001), which are relatively more common in migrants than in non-migrants, and for genitourinary TB (p < 0.001), which is less common in migrants.

**Table 2:** Site of tuberculosis (TB) among migrant and non-migrant extrapulmonary TB^a^ cases in the European Union/European Free Trade Association (EU/EFTA), 1995–2017 (*n* = 251 681)

	Migrants		Non-migrants	
	n	%	n	%
Lymphatic	37 150	47.6∗	43 933	25.3
Pleural	13 730	17.6†	73 052	42.1
Bone/joint incl. spine	6417	8.2∗	12 513	7.2
Disseminated	3947	5.1∗	8245	4.8
Genitourinary	3174	4.1†	11 547	6.7
Peritoneal/digestive	3091	4.0∗	3879	2.2
CNS incl. meningitis	2775	3.6∗	5835	3.4
Other^b^	7793	10.0∗	14 600	8.4
Total^c^	78 077		173 604	

CNS, central nervous system.

∗Proportion is higher in migrants.

†Proportion is higher in non-migrants.

a Extrapulmonary TB is defined as any case of TB involving organs or anatomical sites other than the lungs, with or without coexistent lung disease.

b ‘Other’ refers to TB infection in any organ or anatomical site of the body that falls outside the specified categories.

c There are an additional 100 746 cases not reported here for which the site of TB is known to be extrapulmonary, but the exact site is unknown.

### Extrapulmonary TB by European reporting region

**Fig. 3:**
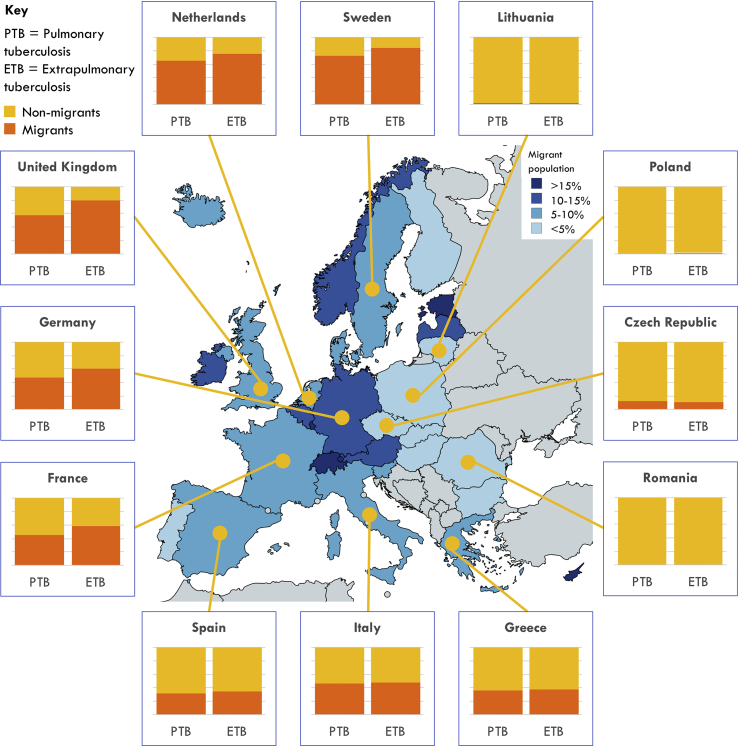
Proportion of pulmonary and extrapulmonary tuberculosis (TB) among migrant and non-migrant TB cases in selected countries of the European Union/European Free Trade Association (EU/EFTA), 1995–2017. Extrapulmonary TB is defined as any case of TB involving organs or anatomical sites other than the lungs, with or without coexistent lung disease. The boxes in the figure illustrate the proportion of pulmonary and extrapulmonary TB cases that occur in migrants in each country. On the map, the different shadings of the countries represent the proportions of individuals with foreign citizenship living in that country on 1st January 2016 (source: Eurostat, online data code: migr_pop1ctz).

A relatively low proportion of extrapulmonary TB is seen in Eastern Europe (17.4%, *n* = 98 656 of 566 170) and Southern Europe (29.6%, *n* = 62 481 of 210 828) compared with Western Europe (35.7%, *n* = 89 498 of 250 517) and Northern Europe (41.8%, *n* = 101 792 of 243 381) (Table 3). Migrants are reported to have a greater proportion of extrapulmonary TB than non-migrants only in Western and Northern Europe (both p < 0.001). For example, in Northern Europe, 58.5% of TB (*n* = 72 868 of 124 547) is extrapulmonary among migrant cases compared with 24.3% (*n* = 28 924 of 118 834) among non-migrants (p < 0.001). Fig. 3 shows that in Northern and Western European countries a high proportion of TB occurs in migrants, with a particularly high proportion of extrapulmonary TB occurring among migrants. In Southern and Eastern European countries, a lower proportion of TB is in migrants, and this pattern does not differ markedly between pulmonary and extrapulmonary TB.

**Table 3:** Site of tuberculosis (TB) among cases reported by countries in the Eastern, Southern, Western and Northern regions of the European Union/European Free Trade Association (EU/EFTA), 1995–2017 (*n* = 1 270 896)

	Pulmonary TB		Extrapulmonary TB^a^		Total
	n	%	n	%	n
**Eastern Europe:**	**467 514**	**82.6**	**98 656**	**17.4**∗	**566 170**
*Migrant*	*3994*	*81.2*	*927*	*18.8*	*4921*
*Non-migrant*	*463 520*	*82.6*	*97 729*	*17.4*	*561 249*
**Southern Europe:**	**148 347**	**70.4**	**62 481**	**29.6**∗	**210 828**
*Migrant*	*47 908*	*69.3*	*21 251*	*30.7*	*69 159*
*Non-migrant*	*100 439*	*70.9*	*41 230*	*29.1*	*141 669*
**Western Europe:**	**161 019**	**64.3**	**89 498**	**35.7**∗	**250 517**
*Migrant*	*75 592*	*58.9*	*52 768*	*41.1*	*128 360*
*Non-migrant*	*85 427*	*69.9*	*36 730*	*30.1*	*122 157*
**Northern Europe:**	**141 589**	**58.2**	**101 792**	**41.8**∗	**243 381**
*Migrant*	*51 679*	*41.5*	*72 868*	*58.5*	*124 547*
*Non-migrant*	*89 910*	*75.7*	*28 924*	*24.3*	*118 834*
**Total**	**918 469**	**72.3**	**352 427**	**27.7**	**1 270 896**

∗ The difference in proportion of TB that is extrapulmonary between each region and each other region is significant at p < 0.001, p values calculated using χ^2^ (e.g. Eastern versus Northern Europe χ^2^ = 5.4 × 10^4^, p < 0.001).

a Extrapulmonary TB is defined as any case of TB involving organs or anatomical sites other than the lungs, with or without co-existent lung disease.

### Extrapulmonary TB and migrants' region of origin

**Fig. 4:**
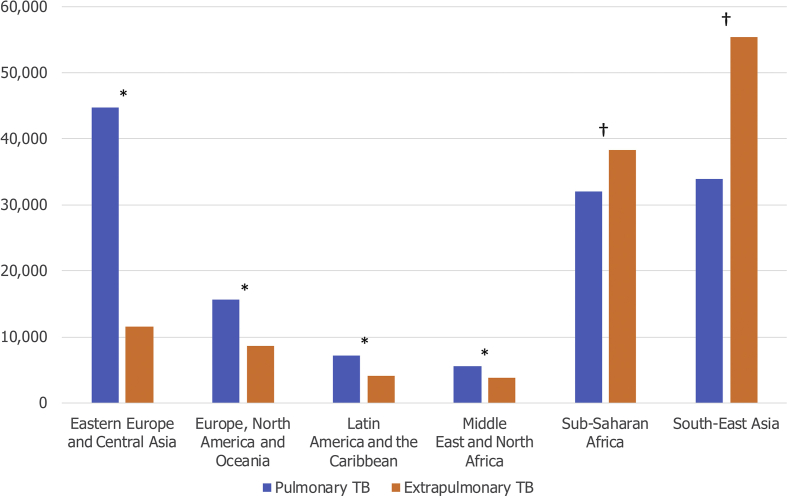
Site of tuberculosis (TB) among migrant TB cases in the European Union/European Free Trade Association (EU/EFTA) by region of origin, 1995–2017 (*n* = 261 074). Extrapulmonary TB is defined as any case of TB involving organs or anatomical sites other than the lungs, with or without coexistent lung disease. ∗p < 0.001, a greater proportion of TB is pulmonary. †p <0.001, a greater proportion of TB is extrapulmonary. p values are two-sided, calculated using one-sample test of proportion (H_0_ = the proportions of pulmonary and extrapulmonary TB are equal).

Over half of TB cases are extrapulmonary among migrants from South-East Asia (62.0%, *n* = 55 401 of 89 353, p < 0.001) and Sub-Saharan Africa (54.5%, *n* = 38 327 of 70 378, p < 0.001) (Fig. 4), which are the most common regions of origin for migrant TB cases in Europe. For example, of 30 174 TB cases among migrants from India, 21 303 (70.6%) were extrapulmonary. In contrast, the proportion of reported extrapulmonary disease is particularly low among migrants from Eastern Europe and Central Asia (20.6%, *n* = 11 576 of 56 310, p < 0.001).

### Sensitivity analyses

In sensitivity analyses, we established that these patterns are seen whether extrapulmonary TB is defined as *any* extrapulmonary or *only* extrapulmonary TB (Supplementary Material Figs S3, S4 and Tables S2–S4).

## Discussion

We found that the proportion of TB that is extrapulmonary is significantly greater among migrants than non-migrants. These data are consistent with single-country studies in the UK and the Netherlands indicating that migrants are at increased risk of extrapulmonary TB [14,15]. The reasons for this are not fully understood. At a time of rising migration to Europe and other high-income countries, with a growing proportion of TB cases occurring in migrants [2,5], extrapulmonary TB in this group is an increasingly important issue. Indeed, the reported proportion of extrapulmonary TB increased from 16.4% in 2002 to 22.4% in 2011 in the EU/EEA [16]. Extrapulmonary TB poses challenges in diagnosis and treatment, with implications for patient outcomes. Thus, migrants' disproportionate burden of extrapulmonary TB may contribute to their worse treatment outcomes [17].

We found that specific site of extrapulmonary TB varies by migrant status. The reasons for this are unclear, but it may reflect the distribution of types of extrapulmonary TB in the countries of origin. For example, India has relatively high rates of lymphatic TB [18], a pattern mirrored among Indian migrants. Migrants were observed to have a lower proportion of pleural TB than non-migrants. However, this should be interpreted with caution, as many cases were reported to include both pulmonary and pleural involvement. Such cases may be classified as having pulmonary plus pleural disease (i.e. extrapulmonary), or as pulmonary disease only. Differences are also seen for other sites of TB (bone/joint, peritoneal/digestive, and genitourinary). However, the absolute numbers of cases are low for these extrapulmonary sites, so a statistical difference may have relatively small clinical implications. These could also potentially be artefacts; for example, genitourinary TB may be misdiagnosed as more common sexually transmitted infections or urinary tract infections, delaying the diagnosis of TB [19].

We also found a higher proportion of extrapulmonary TB in Northern and Western Europe compared with Southern and especially Eastern Europe, with the disproportionate burden of extrapulmonary TB among migrants seen only in Northern and Western Europe. The reasons for this are unclear. Extrapulmonary TB is thought usually to follow reactivation of latent infection rather than occurring at initial infection, with the exception of TB meningitis [20]. Most migrant TB cases are infected before they arrive in host countries, with subsequent reactivation of LTBI [21]. This is especially true in final-destination, high-income countries of Northern and Western Europe, which may contribute to the higher prevalence of extrapulmonary TB in migrants in these regions. Transit countries in Southern and Eastern Europe may see a slightly higher proportion of migrants arriving with active TB or transmission, for example in refugee camps [22]. In addition, healthcare access for migrants varies significantly across Europe [23], and once migrants settle in final-destination countries, they may become better integrated into health systems and more likely to seek care for symptoms of extrapulmonary TB. By contrast, public health initiatives targeting migrants in transit countries tend to focus on preventing the spread of pulmonary TB. However, even in final-destination countries migrants often face significant barriers to healthcare access [24]; therefore, this is unlikely to fully account for the observed differences across Europe.

The proportion of extrapulmonary TB is highest among migrants from South-East Asia and Sub-Saharan Africa. This is consistent with data from Germany and across the EU/EEA showing that migrant TB cases from Asia and Africa were more likely than non-migrant patients to present with extrapulmonary disease [25,26]. Migrants from certain regions may be genetically predisposed to extrapulmonary manifestations of TB [14]. Some countries in Northern and Western Europe host large migrant populations from the Indian subcontinent, where extrapulmonary TB is relatively common [18]. Extrapulmonary TB is also more common in the immunosuppressed, including HIV/AIDS patients [27], and the high burden of TB–HIV co-morbidity in Sub-Saharan Africa may contribute to increased risk of extrapulmonary TB [28]. However, TB–HIV co-morbidity accounts for a small proportion of TB cases in Europe, and is therefore unlikely to be a major driver of the observed patterns.

### Strengths and limitations

By using surveillance data reported by EU/EFTA Member States to ECDC between 1995 and 2017, this study utilizes data from over 1 million TB cases, enabling a comprehensive analysis of patterns of extrapulmonary TB in migrants across Europe. In all EU/EFTA countries, TB is a notifiable disease; however, data quality varies significantly between national surveillance systems, and there are gaps in reporting. We excluded 340 866 TB cases from our analyses because they lacked data on either migrant status or site of TB. The proportion of all notified TB cases that have these data available, and are therefore included, increases over time, from below 50% up to 2001 to over 80% from 2002 and over 90% since 2004 (Supplementary Material Table S5).

Most covariates have good completeness in our dataset (Supplementary Material Table S6). Data on age, gender, and reporting country are over 99% complete, country of origin is 90% complete, previous TB diagnosis is ~85% complete, and site of TB among extrapulmonary cases is ~70% complete. However, HIV status was collected only from 2000 onwards, meaning that in our sample only 17% of cases had data on HIV status. This potentially important variable was therefore excluded from our analyses. The limited nature of the data available prevented us from unpicking the relative impact of host and environmental factors and *Mycobacterium tuberculosis* lineage. This warrants further investigation, as does the pattern of drug resistance in relation to migration status and site of TB. Furthermore, migrant status is defined by country of birth in some countries and country of citizenship in others, which may influence the findings.

### Implications

The findings have important clinical implications for migrant-receiving countries, particularly in Northern and Western Europe. Although there are many reports and guidelines relating to managing TB and migrant health, none adequately addresses the key issues arising from extrapulmonary TB in this group. There is a need to improve diagnostic facilities and awareness of extrapulmonary TB among healthcare staff, particularly when assessing patients from South-East Asia and Sub-Saharan Africa. In addition, detection of extrapulmonary TB should be integrated into screening programmes, with a focus on migrants. Extrapulmonary TB must be considered when screening for latent as well as active disease, when it is essential to ascertain that patients do not have active extrapulmonary disease before initiating preventative chemoprophylaxis for LTBI. Programmes that target migrants for LTBI screening and treatment could therefore have the indirect benefit of detecting and preventing extrapulmonary TB.

## Author contributions

SH, JSF, LBN, and SEH were involved in concept and study design. MvdW and TN facilitated data access and retrieval. SEH carried out the analyses, with input from SH, JSF, LBN and KR. All authors contributed to manuscript writing.

## Transparency declaration

The authors have no conflicts of interest to declare. This work was supported by the 10.13039/501100000265Medical Research Council (MR/N013638/1 to SEH and MR/V027549/1 to LBN), the Rosetrees Trust (M775 to KR), the 10.13039/501100000272National Institute for Health Research (NIHR300072 to SH), the Academy of Medical Sciences (SBF005∖1111 to SH and SBF005∖1047 to LBN), the European Society for Clinical Microbiology and Infectious Diseases (ESCMID Study Group for Infections in Travellers and Migrants (ESGITM)/ESCMID Study Group for Mycobacterial Infection (ESGMYC) research grant to SH), and the Medical Research Council/Economic and Social Research Council/Arts and Humanities Research Council (MR/T046732/1 to LBN).
