# Supplementary material for: Extrapulmonary tuberculosis among migrants in Europe, 1995 to 2017
Source: Clin Microbiol Infect. 2021 Sep;27(9):1347.e1–7. doi: 10.1016/j.cmi.2020.12.006 (PMC8437049; doi:10.1016/j.cmi.2020.12.006)
Supplement: Multimedia component 1 [file mmc1.docx]

**Supplementary Material**

**Figure S1. Definitions**

***Extrapulmonary tuberculosis***: any case of TB involving organs or anatomical sites other than the lungs, with or without co-existent lung disease

***Only extrapulmonary tuberculosis***: a case of TB involving only organs or anatomical sites other than the lungs

***Pulmonary tuberculosis***: a case of TB only involving the lungs

***Migrant***: cases born in a country different to the reporting country (or cases having citizenship different to the reporting country, for cases reported in Austria, Belgium, Greece, and Poland [for all years], Hungary [after 2009], and Malta [before 2007 and in 2010])

***Non-migrant***: cases born in the reporting country (or cases having citizenship of the reporting country, for cases reported in Austria, Belgium, Greece, and Poland [for all years], Hungary [after 2009], and Malta [before 2007 and in 2010])

***Europe***: 32 EU/EFTA countries, as of 2017 (Austria, Belgium, Bulgaria, Croatia, Republic of Cyprus, Czech Republic, Denmark, Estonia, Finland, France, Germany, Greece, Hungary, Iceland, Ireland, Italy, Latvia, Liechtenstein, Lithuania, Luxembourg, Malta, Netherlands, Norway, Poland, Portugal, Romania, Slovakia, Slovenia, Spain, Sweden, Switzerland, and the United Kingdom)

***Eastern Europe****: Bulgaria, Czech Republic, Hungary, Poland, Romania, and Slovakia

***Southern Europe****: Croatia, Republic of Cyprus, Greece, Italy, Malta, Portugal, Slovenia, and Spain

***Western Europe****: Austria, Belgium, France, Germany, Liechtenstein, Luxembourg, Netherlands, and Switzerland

***Northern Europe****: Denmark, Estonia, Finland, Iceland, Ireland, Latvia, Lithuania, Norway, Sweden, and the United Kingdom

EU/EFTA: European Union/European Free Trade Association, TB: tuberculosis

*Defined using the United Nations Geoscheme for Europe

**Figure S2. Region of origin and destination for migrant TB cases in the EU/EFTA, 1995-2017 (n=261,074)**

**
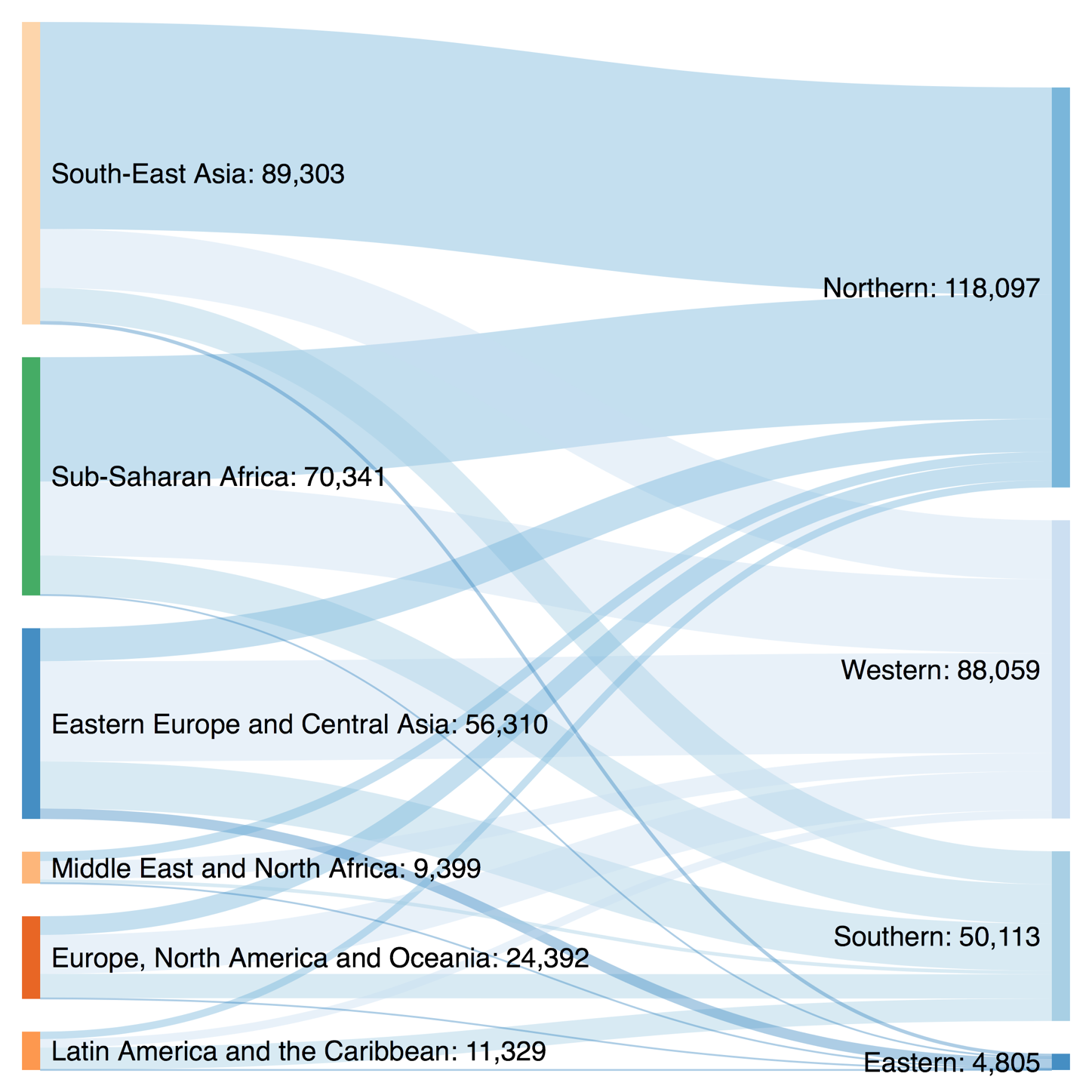
**

**Table S1. Site of TB among migrant and non-migrant TB cases in the EU/EFTA, 1995-2006 (n=521,390) and 2007-2017 (n=749,506)**

|  | **Migrants** | | **Non-migrants** | | **Total** |  |
| --- | --- | --- | --- | --- | --- | --- |
|  | **n** | **%** | **n** | **%** | **n** | **%** |
| 1995-2006 | | | | | | |
| Pulmonary | 67,971 | 56.6 | 312,664 | 77.9 | 380,635 | 73.0 |
| Extrapulmonary | 52,116 | 43.4 | 88,639 | 22.1 | 140,755 | 27.0 |
| 2007-2017 | | | | | | |
| Pulmonary | 111,202 | 53.8 | 426,632 | 78.6 | 537,834 | 71.8 |
| Extrapulmonary | 95,698 | 46.3 | 115,974 | 21.4 | 211,672 | 28.2 |

EU/EFTA: European Union/European Free Trade Association, TB: tuberculosis

**Extrapulmonary tuberculosis defined as a case of TB involving organs or anatomical sites other than the lungs, with or without co-existent lung disease**

The difference between migrants and non-migrants is statistically significant in both time periods at p<0.001 calculated using *χ^2^* test (1995-2006: *χ^2^*=2.1x10^4^; 2007-2017: *χ^2^*=4.6x10^4^)

**Table S2. Site of TB among migrant and non-migrant TB cases in the EU/EFTA, 1995-2017 (n=1,270,809)**

|  | **Migrants** | | **Non-migrants** | | **Total** |  |
| --- | --- | --- | --- | --- | --- | --- |
|  | **n** | **%** | **n** | **%** | **n** | **%** |
| Site of TB | | | | | | |
| Pulmonary | 212,565 | 65.0 | 789,767 | 83.7 | 1,002,332 | 78.9 |
| Extrapulmonary | 114,380 | 35.0 | 154,097 | 16.3 | 268,477 | 21.1 |

EU/EFTA: European Union/European Free Trade Association, TB: tuberculosis

**Extrapulmonary tuberculosis defined as a case of TB involving only organs or anatomical sites other than the lungs**

The difference between migrants and non-migrants is statistically significant at p<0.001 calculated using *χ^2^* test (*χ^2^*=5.1x10^4^)

**Table S3. Site of TB among migrant and non-migrant extrapulmonary TB cases in the EU/EFTA, 1995-2017 (n=185,831)**

|  | **Migrants** | | **Non-migrants** | |
| --- | --- | --- | --- | --- |
|  | **n** | **%** | **n** | **%** |
| Lymphatic | 28,950 | 50.1* | 32,384 | 25.3 |
| Pleural | 9,388 | 16.2† | 52,417 | 41.0 |
| Bone/joint incl. spine | 5,213 | 9.0* | 11,041 | 8.6 |
| Disseminated | 1,351 | 2.3* | 2,473 | 1.9 |
| Genito-urinary | 2,503 | 4.3† | 10,198 | 8.0 |
| Peritoneal/digestive | 2,389 | 4.1* | 2,793 | 2.2 |
| CNS incl. meningitis | 1,981 | 3.4† | 4,735 | 3.7 |
| Other | 6,059 | 10.5* | 11,956 | 9.3 |
| Total | 57,834 | | 127,997 | |

CNS: central nervous system, EU/EFTA: European Union/European Free Trade Association, TB: tuberculosis

**Extrapulmonary tuberculosis defined as a case of TB involving only organs or anatomical sites other than the lungs**

‘Other’ refers to TB infection in any organ or anatomical sites of the body that falls outside the categories above

There are an additional 82,646 cases not reported here for which site of TB is known to be extrapulmonary, but exact site of TB is unknown

* Proportion is higher in migrants

† Proportion is higher in non-migrants

**Table S4. Site of TB among cases in Eastern, Southern, Western and Northern regions of the EU/EFTA, 1995-2017 (n=1,270,809)**

|  | **Pulmonary TB** | | **Extrapulmonary TB** | | | **Total** |
| --- | --- | --- | --- | --- | --- | --- |
|  | **n** | **%** | **n** | | **%** | **n** |
| **Eastern Europe** | **494,133** | **87.3** | **72,037** | **12.7*** | | **566,170** |
| *Migrant* | 4,224 | 85.8 | 697 | 14.2 | | 4,921 |
| *Non-migrant* | 489,909 | 87.3 | 71,340 | 12.7 | | 561,249 |
| **Southern Europe** | **157,290** | **74.6** | **53,522** | **25.4*** | | **210,812** |
| *Migrant* | 50,835 | 73.5 | 18,322 | 26.5 | | 69,157 |
| *Non-migrant* | 106,455 | 75.2 | 35,200 | 24.9 | | 141,655 |
| **Western Europe** | **189,183** | **75.5** | **61,266** | **24.5*** | | **250,449** |
| *Migrant* | 91,465 | 71.3 | 36,858 | 28.7 | | 128,323 |
| *Non-migrant* | 97,718 | 80.0 | 24,408 | 20.0 | | 122,126 |
| **Northern Europe** | **161,726** | **66.5** | **81,652** | **33.5*** | | **243,378** |
| *Migrant* | 66,041 | 53.0 | 58,503 | 47.0 | | 124,544 |
| *Non-migrant* | 95,685 | 80.5 | 23,149 | 19.5 | | 118,834 |
| **Total** | **1,002,332** | **78.9** | **268,477** | **21.1** | | **1,270,809** |

EU/EFTA: European Union/European Free Trade Association, TB: tuberculosis

**Extrapulmonary tuberculosis defined as a case of TB involving only organs or anatomical sites other than the lungs**

*The difference in proportion of TB that is extrapulmonary between each region and each other region is significant at p<0.001, p values calculated using *χ^2^*, e.g. Eastern vs. Northern Europe *χ^2^=*4.8x10^4^, p<0.001

**Figure S4. Site of TB among migrant TB cases in the EU/EFTA by region of origin, 1995-2017 (n=261,034)**

*

†

*

*

*

*

EU/EFTA: European Union/European Free Trade Association, TB: tuberculosis

**Extrapulmonary tuberculosis defined as a case of TB involving only organs or anatomical sites other than the lungs**

*p<0.001, a greater proportion of TB is pulmonary

†<0.001, a greater proportion of TB is extrapulmonary

p values are two-sided, calculated using one-sample test of proportion (H_0_ = the proportions of pulmonary and extrapulmonary TB are equal)

**Table S5. Completeness of included data in comparison with the full TESSy database, 1995-2017**

| **Year** | **Total TB cases** | **TB cases included** | **Completeness (%)** |
| --- | --- | --- | --- |
| 1995 | 47,402 | 7,218 | 15.2 |
| 1996 | 48,778 | 9,917 | 20.3 |
| 1997 | 46,974 | 9,440 | 20.1 |
| 1998 | 54,197 | 15,004 | 27.7 |
| 1999 | 58,875 | 27,918 | 47.4 |
| 2000 | 75,936 | 32,705 | 43.1 |
| 2001 | 86,268 | 39,883 | 46.2 |
| 2002 | 89,153 | 73,814 | 82.8 |
| 2003 | 87,216 | 73,435 | 84.2 |
| 2004 | 84,384 | 81,217 | 96.2 |
| 2005 | 81,264 | 75,992 | 93.5 |
| 2006 | 76,548 | 74,847 | 97.8 |
| 2007 | 84,489 | 81,792 | 96.8 |
| 2008 | 83,597 | 81,230 | 97.2 |
| 2009 | 80,195 | 78,136 | 97.4 |
| 2010 | 76,216 | 74,044 | 97.2 |
| 2011 | 73,903 | 72,169 | 97.7 |
| 2012 | 70,617 | 68,723 | 97.3 |
| 2013 | 66,259 | 62,823 | 94.8 |
| 2014 | 62,313 | 59,446 | 95.4 |
| 2015 | 61,503 | 59,536 | 96.8 |
| 2016 | 59,783 | 57,827 | 96.7 |
| 2017 | 55,892 | 53,780 | 96.2 |
| 1995-2017 | 1,611,762 | 1,270,896 | 78.9 |

TB: tuberculosis, TESSy: The European Surveillance System

‘Total TB cases’ refers to total number of TB cases in the TESSy database in a given year

‘TB cases included’ is the number of TB cases with data on migrant status and site of TB, and therefore included in the analyses

‘Completeness’ is the percentage of all TB cases in TESSy in a given year that are included

**Table S6. Completeness of co-variates in comparison with the included dataset, 1995-2017**

| **Variable** | **Total** | **Missing** | **Completeness (%)** |
| --- | --- | --- | --- |
| Age | 1,270,896 | 2,352 | 99.8 |
| Gender | 1,270,896 | 1,250 | 99.9 |
| Previous TB diagnosis | 1,270,896 | 184,959 | 85.5 |
| Reporting country | 1,270,896 | 0 | 100.0 |
| Country of origin | 1,270,896 | 124,163 | 90.2 |
| Site of extrapulmonary TB | 352,427 | 100,746 | 71.4 |
| HIV | 1,270,896 | 1,051,861 | 17.2 |

TB: tuberculosis, HIV: human immunodeficiency virus

Extrapulmonary TB defined as any case of TB involving organs or anatomical sites other than the lungs, with or without co-existent lung disease

‘Total’ is the number of TB cases included in the analyses, with the exception of ‘site of extrapulmonary TB’, which is restricted to extrapulmonary TB cases

‘Missing’ is the number of TB cases with the listed co-variate missing

‘Completeness’ is the percentage of included TB cases for which the co-variate is available
